# A phase 1 multiple-dose study of orteronel in Japanese patients with castration-resistant prostate cancer

**DOI:** 10.1007/s00280-014-2654-y

**Published:** 2014-12-24

**Authors:** Kazuhiro Suzuki, Seiichiro Ozono, Akito Yamaguchi, Hidekazu Koike, Hiroshi Matsui, Masao Nagata, Takatoshi Takubo, Kana Miyashita, Takafumi Matsushima, Hideyuki Akaza

**Affiliations:** 1 Department of Urology, Gunma University Graduate School of Medicine, Gunma, Japan; 2 Department of Urology, Hamamatsu University School of Medicine, Shizuoka, Japan; 3 Harasanshin Hospital, Fukuoka, Japan; 4 Takeda Pharmaceutical Company Limited, Osaka, Japan; 5 Department of Strategic Investigation on Comprehensive Cancer Network, Research Center for Advanced Science and Technology, The University of Tokyo, Tokyo, Japan

**Keywords:** Orteronel, Castration-resistant prostate cancer, 17,20-Lyase inhibitor, Phase 1

## Abstract

**Purpose:**

Orteronel (TAK-700) is a non-steroidal, selective, reversible inhibitor of 17,20-lyase. We evaluated the safety, tolerability, pharmacokinetics, pharmacodynamics, and antitumor effect of orteronel with or without prednisolone in Japanese patients with castration-resistant prostate cancer (CRPC).

**Methods:**

We conducted a phase 1 study in men with progressive and chemotherapy-naïve CRPC. Patients received orteronel orally at doses of 200–400 mg twice daily (BID) with or without oral prednisolone (5 mg BID). Dose-limiting toxicity (DLT) was assessed during Cycle 1 (28 days). Patients could continue study treatment until any of criteria for treatment discontinuation were met. Gonadotropin-releasing hormone therapy was continued in patients without prior orchidectomy.

**Results:**

Fifteen patients were enrolled and administered at least one dose of orteronel. No DLTs were reported during Cycle 1 in this study. Adverse events (AEs) were reported in all 15 patients. Most common AEs (>30 %) were hyperlipasemia (47 %), hyperamylasemia (40 %), and constipation (33 %). Acute pancreatitis (Grades 2 and 3) and pancreatitis (Grade 1) were complicated in three patients during the study. Dose-dependent increase in plasma orteronel concentrations was indicated over the 200–400 mg BID dose range. Prednisolone coadministered did not alter PK of orteronel. Serum testosterone was rapidly suppressed below the lower limit of quantification across all doses. Of 15 subjects, 13 achieved at least a 50 % reduction from baseline in prostate-specific antigen.

**Conclusions:**

Orteronel at doses up to 400 mg BID was tolerable in Japanese CRPC patients. The present results support further evaluation of orteronel with or without prednisolone.

## Introduction

Prostate cancer is the second most common cancer in men worldwide [1]. In Japan, the number of patients with prostate cancer is rapidly increasing because of the aging of the population, westernization of dietary habits, and advances in diagnostic techniques. The incidence of the malignancy is estimated at 15.1 per 100,000 patients (age-standardized rate) [1].

Castration by orchidectomy or treatment with agonists of receptors for gonadotropin-releasing hormone (GnRH), also known as luteinizing hormone-releasing hormone (LH-RH), has been the standard of care in advanced prostate cancer. Patients with prostate cancer that has recurred after local therapy or has distantly disseminated usually respond to androgen deprivation therapy (ADT); however, most patients eventually experience disease progression within a median of 18–24 months [2].

The intra-prostatic synthesis of testosterone from adrenal-derived precursors likely accounts for the relatively high testosterone levels in the prostate after ADT [3] and castration-resistant prostate cancer (CRPC) cells express all necessary enzymes for the *de novo* synthesis of dihydrotestosterone [4]. CYP17A1 is a key enzyme in the generation of androgens and estrogens in the adrenal glands and tumor tissue, and it catalyzes two independently regulated steroid reactions, involving 17α-hydroxylase and 17,20-lyase in the biosynthesis pathway [5]. This finding led to the concept of CYP17A1 inhibition for depleting both intra-tumoral and extragonadal sources of steroid ligands [5–9]. The importance of this pathway in CRPC has been supported by positive results in phase 3 trials with abiraterone acetate (Zytiga^®^) [10, 11]. However, toxicities attributed to a syndrome of secondary mineralocorticoid excess have been observed with abiraterone, and coadministration of a mineralocorticoid receptor antagonist or glucocorticoid is required to suppress adrenocorticotropic hormone (ACTH) levels [12].

Orteronel (TAK-700) is a non-steroidal, selective, reversible inhibitor of 17,20-lyase. It inhibited 17,20-lyase activity 5.4-fold more potently than it suppressed 17-hydroxylase activity in cell-free enzyme assays, suggesting that orteronel treatment results in the inhibition of androgen synthesis with only partial inhibition of 17α-hydroxylase activity, which allows adrenal cortisol synthesis to continue. Preclinical studies revealed that orteronel reduced serum androgen levels in vivo in monkeys [13]. By selectively inhibiting the extragonadal synthesis of androgens in either the adrenal cortex or prostate tumor cells, orteronel may represent a new therapeutic option for patients with CRPC. The relative selectivity of orteronel for 17,20-lyase may also offer benefits compared with other therapies that target androgen synthesis accompanied by decreased requirements for concurrent administration of steroids such as prednisolone.

This is the first clinical report to evaluate the safety, tolerability, pharmacokinetics (PK), pharmacodynamics (PD), and efficacy of orteronel based on the findings of a phase 1 study in Japanese patients with chemotherapy-naïve CRPC.

## Patients and methods

### Patients

Patients were recruited from three centers in Japan. The study was conducted in accordance with the Declaration of Helsinki/Good Clinical Practices. The Institutional Review Board approved all aspects of the study, and all participants provided written informed consent. The eligibility criteria included histologically or cytologically confirmed prostate adenocarcinoma, prostate-specific antigen (PSA) levels of ≥2 ng/mL at screening, an increase from nadir in PSA levels in ≥2 successive measurements by the time of screening, well-controlled castration (serum testosterone level <0.5 ng/mL), an Eastern Cooperative Oncology Group (ECOG) performance status of 0–2 at screening, and adequate liver, renal, and bone marrow functions. Patients were also required to discontinue all antiandrogen therapy for at least 6 (bicalutamide) or 4 weeks (any other therapy) before the first dose of orteronel. Patients with an unacceptable cardiac complication were excluded. Patients who had received prior chemotherapy for prostate cancer were also excluded. Eligibility did not require a determination of the presence or absence of metastasis.

### Study design

This was a phase 1, open-label, multiple-dose study in Japanese patients with chemotherapy-naïve, hormone therapy-resistant prostate cancer (also known as CRPC). For patients who had not undergone prior orchidectomy, the castrated testosterone level was maintained by GnRH therapy. The primary objective of this study was to assess the safety, tolerability, and PK of orteronel as a single agent or in combination with prednisolone in patients with CRPC. The secondary objective was to determine the PD and antitumor effect of orteronel using serum PSA levels, PSA response rates, and objective disease response rates in patients with CRPC [14].

Patients received orteronel orally either with or without oral prednisolone at the following doses: 200 mg twice daily (200-mg BID cohort), 400 mg BID (400-mg BID cohort), or 400 mg BID plus prednisolone 5 mg BID (400-mg BID plus prednisolone cohort). The propriety of transitions to subsequent dose cohort was judged on the basis of the number of patients with dose-limiting toxicities (DLTs) during Cycle 1 (28 days). Furthermore, additional two cohorts in which subjects received orteronel 300 mg BID (300-mg BID cohort) or orteronel 300 mg BID plus prednisolone 5 mg BID (300-mg BID plus prednisolone cohort) were assessed on the basis of a preliminary interim analysis. Patients could continue study treatment until criteria for treatment discontinuation were met. The use of aldosterone antagonists (e.g., eplerenone) was allowed for patients with clinical symptoms of mineralocorticoid excess such as hypertension, hypokalemia, and edema.

### Assessments

Safety assessments included the evaluation of adverse events (AEs), laboratory profiles, physical examination, and vital signs throughout the study. AEs were graded using National Cancer Institute Common Terminology Criteria for Adverse Events (NCI-CTCAE) v3.0. Analysis of serum amylase and lipase was added to the clinical laboratory tests before enrollment of patients into the 400-mg BID plus prednisolone cohort because pancreatitis was reported during the study. A safety follow-up was performed for 28 days after the final dose of the study treatment. During Cycle 1 (28 days), DLT was also evaluated. DLTs were defined as any drug-related AE of ≥ Grade 3 and pre-specified cardiac-related AEs of lower grade. Hypertension, hypokalemia, and edema attributed to increased ACTH secretion maintained at Grade 2 or lower through the use of eplerenone were not considered DLTs.

The pharmacokinetic endpoints were to evaluate PK profiles in plasma and urinary excretion of unchanged orteronel and a primary metabolite (M-I). Unchanged orteronel and M-I in plasma and urine were determined by the validated high-performance liquid chromatography–tandem mass spectrometry.

Endocrine parameters such as serum testosterone and dehydroepiandrosterone sulfate (DHEA-S) were measured for PD analysis. The blood sample were collected on Days 1, 8, 15, and 22 of Cycle 1 and then on Day 1 of each subsequent 28-day cycle. Assay procedure for testosterone was improved during the study, and the lower limit of quantification (LLOQ) for testosterone changed from 0.05 to 0.03 ng/mL.

The efficacy endpoints were serum PSA levels, PSA response rates based on the Prostate Cancer Clinical Trials Working Group (PCWG) 2 criteria, and objective disease response rates based on the General Rule for Clinical and Pathological Studies on Prostate Cancer and modified RECIST version 1.0. PSA levels were measured on Days 1 and 15 in Cycle 1, on Day 1 of Cycle 2 and each subsequent cycle. Tumor assessments were performed on Day 29, 85, and every 12-week cycle for subsequent cycles.

### Statistical analysis

Three patients (a maximum of six patients) were to be included in each cohort. The number of patients was determined according to the “Guidelines on clinical evaluation method of antineoplastics” in Japan (PFSB/ELD, 2005) [15], and the setting was not based on the statistical rationale. Standard summary statistics were used for safety, PK, PD, and efficacy analyses.

## Results

### Patients and exposure

Between April 2010 and January 2012, 15 patients (3 in each cohort) were enrolled in this study. All 15 patients received at least one dose of orteronel. At the time of analysis, 13 patients had discontinued orteronel treatment because of the following reasons: lack of efficacy in eight patients and TEAEs in five patients. The remaining two patients were under treatment: One patient in the 200-mg BID cohort and one patient in the 400-mg BID plus prednisolone cohort whose current dose of orteronel was reduced to 200 mg BID with prednisolone 5 mg BID.

Demographic and other baseline characteristics are shown in Table 1. The overall median age was 71.0 years. All 15 patients experienced relapses and had a baseline ECOG performance status of 0. Gleason scores at the time of the latest examination were 7 or more for all patients. All patients received prior medication for prostate cancer, and two patients (13 %) received other prior therapies for prostate cancer including radiation to prostate gland in one patient and palliative radiation to lumbar vertebrae metastasis and bilateral orchidectomy in one patient.

**Table 1:** Demographic and other baseline characteristics

	Orteronel
	All subjects (*N* = 15)
Sex—*n* (%)	
Male	15 (100)
Age—years	
*n*	15
Mean (SD)	69.5 (4.90)
Median	71.0
Min, max	60, 77
Gleason classification score at the time of the latest examination—*n* (%)	
≤6	0 (0)
7	1 (7)
8	4 (27)
9	7 (47)
10	3 (20)
Histological type—*n* (%)	
Adenocarcinoma	14 (93)
Well differentiated	0 (0)
Moderately differentiated	3 (20)
Poorly differentiated	10 (67)
Unclassified	1 (7)
Other	1 (7)
TNM classification—*n* (%)	
T3N0M0	2 (13)
T3N0M1	2 (13)
T3N1M0	2 (13)
T3N1M1	2 (13)
T3N1MX	1 (7)
T4N0M1	2 (13)
T4N1M1	3 (20)
TXN0M1	1 (7)
Eastern cooperative oncology group (ECOG) performance status—*n* (%)	
0	15 (100)
1 or 2	0 (0)

The median duration of treatment days across the cohorts was 276.0 days, and the treatment duration was shorter in the 400-mg BID cohort (60.0 days) than in the other cohorts (Table 2). All 15 patients completed Cycle 1 treatment at the prescribed dose and were eligible for DLT assessment.

**Table 2:** Exposure to orteronel

	Orteronel					
	200 mg (*N* = 3)	300 mg (*N* = 3)	300 mg + prednisolone (*N* = 3)	400 mg (*N* = 3)	400 mg + prednisolone (*N* = 3)	All subjects (*N* = 15)
Treatment days (days)						
*n*	3	3	3	3	3	15
Mean (SD)	597.0 (307.44)	342.7 (129.07)	172.3 (92.32)	61.0 (24.52)	397.3 (311.21)	314.1 (260.51)
Median	420.0	399.0	142.0	60.0	499.0	276.0
Min, max	419, 952	195, 434	99, 276	37, 86	48, 645	37, 952
Total amount of doses taken (10^3^ mg)						
*n*	3	3	3	3	3	15
Mean (SD)	227.4 (133.89)	171.5 (63.49)	86.9 (49.69)	36.7 (8.93)	156.1 (131.92)	135.7 (103.74)
Median	167.4	156.2	82.5	35.0	133.0	133.0
Min, max	134, 381	117, 241	40, 139	29, 46	37, 298	29, 381
Relative dose intensity^a^ in cycle 1 (%)						
*n*	3	3	3	3	3	15
Mean (SD)	100.00 (0.000)	100.00 (0.000)	99.80 (0.344)	95.24 (8.248)	100.00 (0.000)	99.01 (3.681)
Median	100.00	100.00	100.00	100.00	100.00	100.00
Min, max	100.0, 100.0	100.0, 100.0	99.4, 100.0	85.7, 100.0	100.0, 100.0	85.7, 100.0
Relative dose intensity^a^ during study (%)						
*n*	3	3	3	3	3	15
Mean (SD)	93.21 (11.650)	85.96 (18.311)	82.34 (15.212)	79.22 (15.894)	65.77 (36.373)	81.30 (20.331)
Median	99.88	92.63	83.70	72.92	74.65	83.70
Min, max	79.8, 100.0	65.2, 100.0	66.5, 96.8	67.4, 97.3	25.8, 96.9	25.8, 100.0

^a^Relative dose intensity = (total dose received/total dose expected per initial dose) × 100

### Safety results

The treatment-emergent AEs (TEAEs) that occurred in ≥10 % of patients during the study are shown in Table 3. Neither DLTs nor serious AEs (SAEs) were reported in Cycle 1 in any cohort. Overall, all 15 patients (100 %) experienced at least 1 TEAE, and 14 patients (93 %) experienced at least 1 treatment-related AE. Most common TEAEs that were observed in >30 % of patients were hyperlipasemia, hyperamylasemia, and constipation.

**Table 3:** Subject incidence of treatment-emergent adverse events by preferred term in descending order of frequency (≥10 % subjects in all subjects during the study, any Grade and Grade 3 or higher)

Preferred term	TAK-700 (orteronel)											
	200 mg (*N* = 3) *n* (%)		300 mg (*N* = 3) *n* (%)		300 mg + prednisolone (*N* = 3) *n* (%)		400 mg (*N* = 3) *n* (%)		400 mg + prednisolone (*N* = 3) *n* (%)		All subjects (*N* = 15) *n* (%)	
	Any grade	Grade 3 or higher	Any grade	Grade 3 or higher	Any grade	Grade 3 or higher	Any grade	Grade 3 or higher	Any grade	Grade 3 or higher	Any grade	Grade 3 or higher
Any adverse event												
Number of subjects	3 (100)	1 (33)	3 (100)	3 (100)	3 (100)	3 (100)	3 (100)	2 (67)	3 (100)	3 (100)	15 (100)	12 (80)
Hyperlipasemia	–	–	3 (100)	3 (100)	3 (100)	2 (67)	1 (33)	1 (33)	–	–	7 (47)	6 (40)
Hyperamylasemia	–	–	2 (67)	1 (33)	3 (100)	1 (33)	1 (33)	1 (33)	–	–	6 (40)	3 (20)
Constipation	–	–	–	–	2 (67)	–	–	–	3 (100)	–	5 (33)	–
Dysgeusia	–	–	–	–	1 (33)	–	3 (100)	–	–	–	4 (27)	–
Hepatic function abnormal	1 (33)	–	1 (33)	–	–	–	1 (33)	–	1 (33)	–	4 (27)	–
Hypertension	1 (33)	–	1 (33)	1 (33)	–	–	2 (67)	–	–	–	4 (27)	1 (7)
Malaise	–	–	–	–	1 (33)	–	2 (67)	–	1 (33)	–	4 (27)	–
Hypokalemia	–	–	–	–	–	–	3 (100)	1 (33)	–	–	3 (20)	1 (7)
Nasopharyngitis	1 (33)	–	–	–	–	–	–	–	2 (67)	–	3 (20)	–
Nausea	–	–	1 (33)	–	–	–	1 (33)	–	1 (33)	–	3 (20)	–
Rash	–	–	–	–	–	–	–	–	3 (100)	–	3 (20)	–
Abdominal pain upper	–	–	–	–	–	–	1 (33)	–	1 (33)	–	2 (13)	–
Blood creatine phosphokinase increased	1 (33)	–	–	–	1 (33)	–	–	–	–	–	2 (13)	–
Contusion	–	–	–	–	–	–	1 (33)	–	1 (33)	–	2 (13)	–
Diabetes mellitus	–	–	–	–	1 (33)	1 (33)	–	–	1 (33)	–	2 (13)	1 (7)
Fall	–	–	–	–	–	–	–	–	2 (67)	–	2 (13)	–
Insomnia	1 (33)	–	–	–	–	–	–	–	1 (33)	–	2 (13)	–
Pancreatitis acute	–	–	–	–	–	–	1 (33)	–	1 (33)	1 (33)	2 (13)	1 (7)
Pneumonia	–	–	–	–	1 (33)	–	–	–	1 (33)	1 (33)	2 (13)	1 (7)
Vomiting	–	–	–	–	–	–	1 (33)	–	1 (33)	–	2 (13)	–

Adverse events were coded using the MedDRA version 13.0

A subject counts once for each preferred term

No deaths were reported in this study. Nine patients (60 %) experienced a total of 14 SAEs, of which nine were considered to be related to the study treatment. No treatment-related SAE was observed in the 200- and 300-mg BID cohorts. Acute pancreatitis was reported in two patients (400-mg BID cohort and 400-mg BID plus prednisolone cohort) and resulted in permanently discontinuation of orteronel. Other treatment-related SAEs reported in one patient each were diabetes mellitus, gastric ulcer hemorrhage, hyperamylasemia, hyperlipasemia, interstitial lung disease, pneumonitis, and pyrexia. We had two experiences of the elevation of pancreatic enzymes in 400-mg BID cohort. First one was observed on Day 60. The patient had complaint of continuous abdominal discomfort. Laboratory data of amylase and lipase were elevated. Slight peripancreatic effusion was pointed out by abdominal CT scan. The signs and symptom were diagnosed as acute pancreatitis. After discontinuation of orteronel, the symptom and abnormal data were resolved. Second one was asymptomatic and checked in some laboratory data including amylase and lipase as monitoring. The patient had Grade 3 hyperamylasemia and Grade 4 hyperlipasemia with unremarkable pancreatic change by CT scan. Afterward, we added the periodical monitoring of pancreatic enzymes in this protocol after these events. The onset of abnormal pancreatic enzymes was reported during early treatment phase (around Cycle 2 or Cycle 3). In most patients, these abnormal elevations were reported without any symptom or radiographic abnormalities in pancreas. Three conditions were diagnosed as acute pancreatitis or pancreatitis in this study. Other two pancreatitis were also reported in 400-mg BID cohort with or without prednisolone (Grades 1 and 2). In 300-mg BID orteronel with or without prednisolone cohorts, pancreatitis was not reported. A total of ten patients experienced pancreatic-related AEs. Two patients were recovered from the abnormality without any dose change of orteronel. Other three patients were rechallenged with orteronel after the pancreatic enzyme returned to normal. Of these three patients, two continued treatment without any experience of pancreas-related TEAEs. Another patient experienced a re-elevation of pancreatic enzyme, but pancreatitis was not complicated.

Mineralocorticoid-related TEAEs such as hypertension (*n* = 4) and hypokalemia (*n* = 3) were mild, and the symptoms were manageable without dose reduction or dose hold of orteronel except for one Grade 3 hypokalemia. All patients who experienced these events were enrolled in prednisolone-free cohort. No TEAE associated with adrenal insufficiency was reported in this study.

No Grade 3 or higher abnormalities were observed in hematologic test or urinalysis. No clinically significant abnormal 12-lead ECG and echocardiography findings were observed either in any patient or at any assessment point during the study.

### PK results

The maximum plasma concentration (*C*_max_) of orteronel was observed at approximately 1–3 h after the morning dose on Day 8 of Cycle 1 in all cohorts (Table 4). The plasma concentrations of orteronel increased with the BID dosing in all cohorts and the cumulative ratios of area under the plasma concentration–time curve (AUC) on Day 8 ranged from 1.10 to 1.67. Dose-dependent increases in *C*_max_ and AUC of orteronel on Day 8 were indicated in the dose range of the 200 and 400 mg BID. Differences in the values of PK parameters between prednisolone-free and prednisolone coadministration were not clearly observed at orteronel doses of 300 and 400 mg BID. The time to achieve *C*_max_ (*T*_max_) of M-I on Day 8 was approximately 3–8 h in all cohorts. AUC of M-I on Day 8 was approximately 30–50 % of that of orteronel in each cohort. The fractional urinary excretion rates for 24 h after the morning dose on Day 15 ranged from 36 to 47 % of the dose for orteronel and from 14 to 21 % of the dose for M-I, indicating that more than half of the oral dose was excreted into urine in all cohorts.

**Table 4:** Pharmacokinetic parameters of orteronel and M-I on Day 8 of Cycle 1

PK parameter	Cohort				
	200 mg BID	300 mg BID	300 mg + pred BID	400 mg BID	400 mg + pred BID
Orteronel					
*T*_max_	1.00 (1.00, 2.92)	3.00 (2.00, 7.75)	3.00 (2.00, 5.00)	2.97 (2.95, 2.98)	2.00 (1.08, 3.00)
*C*_max_ (μg/mL)	1.58 (0.38)	3.10 (1.0)	3.19 (0.42)	4.70 (1.3)	5.96 (2.1)
AUC_0–8h_ (h μg/mL)	7.64 (2.1)	14.5 (4.5)	16.7 (1.4)	28.6 (11)	30.7 (11)
R (AUC)	1.58 (0.62)	1.10 (0.17)	1.67 (0.33)	1.46 (0.42)	1.66 (0.13)
M-I					
*T*_max_	2.92 (2.00, 5.00)	7.75 (5.00, 8.00)	4.98 (3.00, 5.00)	4.83 (2.98, 4.93)	3.00 (2.75, 5.00)
*C*_max_ (μg/mL)	0.478 (0.25)	0.885 (0.27)	1.25 (0.21)	1.37 (0.37)	1.47 (0.67)
AUC_0–8h_ (h μg/mL)	3.17 (1.5)	5.29 (1.2)	7.97 (2.0)	9.02 (1.6)	9.83 (4.2)

*n* = 3, median (min, max) for *T*_max_, mean (SD) for other parameters

AUC_0–8h_: area under the plasma concentration–time curve from 0 to 8 h post-dose, *C*_max_: maximum plasma concentration, pred: 5 mg prednisolone, R (AUC): AUC_0–8h_ [Day 8]/AUC_0–8h_ [Day 1], *T*_max_: time to *C*_max_

### PD and antitumor effects

The endocrine parameters are presented in Table 5. Serum testosterone was suppressed to LLOQ or lower by 1 week after the initiation of orteronel treatment (Day 8) across all cohorts. Serum DHEA-S was also suppressed by orteronel on Day 8. The mean level of serum DHEA-S on Day 8 was lower in 400 mg BID of orteronel than lower dose of orteronel. In addition, the levels in prednisolone-coadministration cohorts were lower than those in corresponding prednisolone-free cohorts.

**Table 5:** Summary statistics of endocrine panel

Assessment (unit) visit		Orteronel				
		200 mg (*N* = 3)	300 mg (*N* = 3)	300 mg + prednisolone (*N* = 3)	400 mg (*N* = 3)	400 mg + prednisolone (*N* = 3)
Testosterone (ng/mL)						
Screening	Mean (SD)	0.153 (0.0611)	0.123 (0.0702)	0.127 (0.0473)	0.057 (0.0115)	0.053 (0.0058)
Cycle 1 Day 8	Mean (SD)	0.050 (0.0000)	0.030 (0.0000)	0.030 (0.0000)	0.050 (0.0000)	0.043 (0.0115)
Cycle 2 Day 1	Mean (SD)	0.050 (0.0000)	0.030 (0.0000)	0.030 (0.0000)	0.050 (0.0000)	0.037 (0.0115)
DHEA-S (μg/dL)						
Screening	Mean (SD)	114.7 (8.96)	146.7 (79.83)	136.0 (52.68)	60.3 (8.50)	40.3 (7.57)
Cycle 1 Day 8	Mean (SD)	25.0 (7.21)	23.3 (14.57)	6.3 (4.16)	9.3 (0.58)	2.3 (0.58)
Cycle 2 Day 1	Mean (SD)	30.0 (9.64)	24.3 (11.93)	2.3 (0.58)	19.3 (16.29)	2.0 (0.00)

The lower limits of quantification (LLOQ) are: testosterone 0.05 and 0.03 ng/dL, DHEA-S 2.0 μg/dL. Testosterone assay was changed during the study, making LLOQ changed from 0.05 to 0.03 ng/mL

A cycle was defined as a 4-week (28-day) period

*DHEA*-*S* dehydroepiandrosterone sulfate

The overall median maximum PSA reduction was 88.48 % during the study. Of 15 PSA-evaluable patients, 13 (87 %) achieved at least a 50 % reduction from baseline in PSA. With regard to objective disease responses based on the General Rule for Clinical and Pathological Studies on Prostate Cancer, no patient had a best response of complete response (CR) and five patients had a best response of partial response (PR). The objective response rate (CR + PR) was 33 % (5/15 patients). Regarding objective disease responses based on modified RECIST version 1.0, only six patients had measurable disease. No patient had a best response of CR or PR.

## Discussion

A total of 15 patients were treated by orteronel at doses of 200, 300, 300 mg BID plus prednisolone, 400, and 400 mg BID plus prednisolone. Orteronel was well tolerated at doses up to 400 mg BID in Japanese CRPC patients either as a single agent or in combination with prednisolone. No DLTs were reported during the DLT assessment period in any cohort. PSA responses and testosterone levels indicated the potential clinical efficacy of orteronel with or without prednisolone coadministration.

The small amount of remaining androgens after castration for prostate cancer plays an important role in the proliferation of prostate tumor cells. Abiraterone was developed as an androgen production inhibitor [11]. To prevent mineralocorticoid excess syndrome and adrenal insufficiency, prednisolone is required to coadminister with abiraterone. Orteronel selectively inhibits 17,20-lyase and may offer benefits compared with other therapies that target androgen synthesis, with a reduced need for concurrent administration of steroids (prednisolone). In contrast, high dose of orteronel may block 17-hydroxylase activity, thereby resulting in increased mineralocorticoid activity in patients not receiving concomitant glucocorticoids. Thus, mineralocorticoid excess syndrome (hypertension and hypokalemia) and adrenal insufficiency-related TEAEs were assessed as significant AEs in this study.

Consequently, as mineralocorticoid excess syndrome-related TEAEs, hypertension, and hypokalemia were reported. Both events were observed in the prednisolone-free cohorts, and most patients who experienced these events received high dose (400 mg BID) of orteronel. These patients except one did not require dose reduction or dose hold, and their symptoms were manageable by treatment with eplerenone and other agents. No adrenal insufficiency-related TEAEs were reported in any cohorts. These results suggest that concomitant administration of prednisolone (5 mg BID) may not be required for preventing mineralocorticoid excess syndrome-related TEAEs, especially in lower doses of orteronel (200 or 300 mg BID). Pancreatic enzyme abnormalities were observed with relatively high frequencies. Such changes were asymptomatic in most patients, who did not display the evidence of pancreatitis in diagnostic imaging. In the study, increases in pancreatic enzyme levels were intensively reported in Cycles 2 and 3, and these abnormalities subsequently returned to normal without dose change or after dose discontinuation of orteronel. Although some patients resumed orteronel after recovering from the abnormality, no one complicated acute pancreatitis by rechallenge of orteronel. The patients who received orteronel for a long period did not exhibit an abnormal pancreatic enzyme in the late treatment phase (Day 150 or later), suggesting that increases in pancreatic enzyme levels occurred in the early treatment phase of orteronel, and that the possibility of delayed and/or recurrent increases is relatively low. In the case of asymptomatic elevation of pancreatic enzymes, the abnormalities were reversible by dose hold of orteronel, and the orteronel treatment could be resumed with or without dose reduction. Although the mechanism(s) of the increases in pancreatic enzymes is unclear, it is possible that orteronel causes drug-induced pancreatic hyperenzymemia rather than pancreatitis similarly as other drugs such as sorafenib [16]. We need to further discuss the relationship between increase in pancreatic enzyme and in pancreatitis.

*T*_max_ of orteronel was approximately 1–3 h after oral administration, and more than half of the oral dose was excreted into urine in all cohorts. M-I was considered to be a primary metabolite of orteronel, judging from the AUC ratio of M-I to orteronel and the urinary excretion rate of M-I. These findings indicated that orteronel is well absorbed after oral administration, and absorbed orteronel is primarily metabolized into M-I as well as urinary excretion. The comparison of the values of PK parameters between prednisolone-free and prednisolone coadministration suggested no remarkable effect of coadministered prednisolone (5 mg) on the PK of orteronel.

Serum testosterone was rapidly suppressed to the LLOQ or lower across all cohorts, suggesting the expected pharmacological effects by orteronel in Japanese CRPC patients. Serum DHEA-S, which is the upstream hormone of testosterone synthesis, was also suppressed by orteronel administration. The reduction in serum DHEA-S levels appeared to depend on the orteronel dosage or concurrent administration of prednisolone. However, because of the sensitivity of hormone assays and degree of variability among patients, no significant differences were found in endocrine responses between the 300- and 400-mg BID orteronel. Due to the small number of patients in this study, it may require further evaluation to determine whether 300- or 400-mg BID orteronel is recommended for Japanese CRPC patients.

Mean and median reductions in PSA were evaluated during the study. However, the number of patients was too small to draw meaningful conclusions regarding the dose. The compliance of 2 PSA non-responders to orteronel dosing was sufficient during the study according to their relative dose intensities (96.8 and 83.7 %, respectively), and their serum testosterone and DHEA-S concentrations were suppressed to the LLOQ or lower. It has been reported that a certain type of prostate cancer cells grows in a testosterone-independent manner [17], and thus, the 2 PSA non-responders may have developed a testosterone-independent type of prostate cancer.

In conclusion, orteronel at doses up to 400 mg BID was tolerable in Japanese CRPC patients. The results of the present study support further evaluation of orteronel with or without prednisolone.
